# Congestion

**Published:** 1896-01

**Authors:** E. S. Collier

**Affiliations:** Wills Point, Texas


### Congestion.

WILLS POINT, TEXAS, Nov. 2, 1895.

*Editors Texas Medical Journal:*

There is a condition with which we are meeting, charcterized as, or termed, "congestion," by the busy practitioner. Probably the history runs about like this: The patient has a history of a possible chill, or some little fever of a few hours duration, may be not even that, for a few days previous; then he has a fever to come rapidly, with or without the chill; then he begins to have some labored breathing; you find him bathed in perspiration, with high fever—often very little fever; he has the breathing characteristic of imperfectly oxygenated blood; his pulse is hardly perceptible, going at the rate of 120 to 160 to the minute. In other words, he is suffering from the effects of "pernicious malaria," and the friends are crying out "congestive chill!" et cetera.

I am now theorizing. My object in writing you is to ask your opinion as to the actual pathology of the term "congestion," as applied to such a class of cases. Is it an actual venous stasis, capillary anæmia? If so, is it caused by heart weakness?

Also as to its treatment: We are in the habit of letting some of such cases die. May be they are walking about in the morning, take a chill, or a fever without the chill, and you are sent for in time to see them in a dying condition. Such is hardly an epidemic; our community has lost several persons in that way lately.

Our practice is to administer quinine heavily, atropine, morphine, digitalis; wrap in hot blankets or sheets, use ammonia, and any or all heart stimulants. Please "dilate" a little upon the actual condition, pathological, of that kind of "congestion"; also state whether or not you consider pilocarpine indicated in such a condition, if you understand my case well enough from my hurried description. Such might give immediate relief while awaiting the effect of a thirty-grain dose of quinine.

Yours, E. S. COLLIER, M. D.

P. S.—If I am impertinent, beg your pardon; but I would like to hear from your journal concerning the above. For a congested stomach, when overloaded, we can unload it; but what I specially wish to know is, what is congested in these malarial troubles, unless it is the spleen, and how to give relief.

E. S. C.

[The doctor's inquiries are answered in our editorial columns by Associate Editor Prof. A. J. Smith, M. D., of the Texas University, Medical Department.—ED.]

